# The Anticancer Drug 3-Bromopyruvate Induces DNA Damage Potentially Through Reactive Oxygen Species in Yeast and in Human Cancer Cells

**DOI:** 10.3390/cells9051161

**Published:** 2020-05-08

**Authors:** Magdalena Cal, Irwin Matyjaszczyk, Ireneusz Litwin, Daria Augustyniak, Rafał Ogórek, Young Ko, Stanisław Ułaszewski

**Affiliations:** 1 Department of Mycology and Genetics, Institute of Genetics and Microbiology, University of Wroclaw, 51-148 Wroclaw, Poland; irwin.matyjaszczyk@uwr.edu.pl (I.M.); rafal.ogorek@uwr.edu.pl (R.O.); stanislaw.ulaszewski@gmail.com (S.U.); 2 Department of Genetics and Cellular Physiology, Institute of Experimental Biology, University of Wroclaw, 50-328 Wroclaw, Poland; ireneusz.litwin@uwr.edu.pl; 3 Department of Pathogen Biology and Immunology, Institute of Genetics and Microbiology, University of Wroclaw, 51-148 Wroclaw, Poland; daria.augustyniak@uwr.edu.pl; 4 KoDiscovery, LLC, UM BioPark, Baltimore, MD 21201, USA; kocancer212@yahoo.com

**Keywords:** 3-bromopyruvate, DNA damage, DNA double-strand break, oxidative stress, yeast, human monocytes

## Abstract

3-bromopyruvate (3-BP) is a small molecule with anticancer and antimicrobial activities. 3-BP is taken up selectively by cancer cells’ mono-carboxylate transporters (MCTs), which are highly overexpressed by many cancers. When 3-BP enters cancer cells it inactivates several glycolytic and mitochondrial enzymes, leading to ATP depletion and the generation of reactive oxygen species. While mechanisms of 3-BP uptake and its influence on cell metabolism are well understood, the impact of 3-BP at certain concentrations on DNA integrity has never been investigated in detail. Here we have collected several lines of evidence suggesting that 3-BP induces DNA damage probably as a result of ROS generation, in both yeast and human cancer cells, when its concentration is sufficiently low and most cells are still viable. We also demonstrate that in yeast 3-BP treatment leads to generation of DNA double-strand breaks only in S-phase of the cell cycle, possibly as a result of oxidative DNA damage. This leads to DNA damage, checkpoint activation and focal accumulation of the DNA response proteins. Interestingly, in human cancer cells exposure to 3-BP also induces DNA breaks that trigger H2A.X phosphorylation. Our current data shed new light on the mechanisms by which a sufficiently low concentration of 3-BP can induce cytotoxicity at the DNA level, a finding that might be important for the future design of anticancer therapies.

## 1. Introduction

3-bromopyruvate (3-BP) is an analog of pyruvic acid that exhibits strong cytotoxic activity towards most cancer cells [1,2,3]. In human cancer cells, 3-BP is taken up by mono-carboxylate transporters (MCTs) [4,5], while in yeast 3-BP enters the cell through the lactate/pyruvate H^+^ symporter Jen1 [6]. Inside the cell 3-BP becomes highly reactive, showing strong alkylating properties towards proteins, metabolites and DNA. It was shown that 3-BP inhibits key glycolytic enzymes including hexokinase II [7,8,9,10], glyceraldehyde 3-phosphate dehydrogenase (GAPDH) [11] and lactate dehydrogenase (LDH) [12]. Furthermore, 3-BP has also been reported to affect mitochondrial functions by inhibiting complexes I and II but not IV [13]. As a consequence, 3-BP treatment leads to massive ATP depletion [13] and increased levels of reactive oxygen species (ROS) [14,15,16,17] along with strong reduction in free glutathione levels [18,19,20,21]. Interestingly, because many cancer cells rely on glycolysis as a source of ATP and thus overproduce MCTs to efflux excess lactic acid [22], concentrations of 3-BP that are neutral for normal cells are strongly toxic to tumor cells, making 3-BP a promising candidate for anticancer therapy [23].

It seems that the molecular action of 3-BP may be highly pleiotropic in the cell [1,3,24]. For the first time, antitumor properties of 3-BP were discovered during studies using animal models [25]. Subsequently, studies on multiple myeloma cancer cell lines (MM cells) also helped to better understand 3-BP’s anticancer mechanism [2]. More recently, it has been demonstrated in *Cryptococcus neoformans* and MM that reactive oxygen species (ROS) are formed as a result of 3-BP treatment [20,26,27]. Finally, studies on human cell lines as well as on fungal and algal cells revealed that glutathione levels decrease upon 3-BP treatment [20,21], which is probably a result of glutathione-3-BP complex formation [19].

All organisms are constantly exposed to a variety of physical and chemical agents that damage DNA and threaten genome stability. To preserve genome integrity, all eukaryotic cells have evolved DNA damage response mechanisms that sense and repair DNA damage including DNA damage checkpoint (DDC) and DNA damage repair mechanisms. In both yeast and mammals, DNA double-strand breaks (DSBs) are first recognized and bound by Mre11-Rad50-Xrs2 (MRX) complex (MRE11-RAD50-NBS1 in humans) that immediately recruits nonessential Tel1 DDC sensor kinase (ATM in humans) [28]. Next, Tel1 phosphorylates histone H2A on serine 129 (H2A-P) in the vicinity of DSB [29] (serine 139 on H2A.X in humans). This allows recruitment of the Rad9 (53BP1, MDC1 in humans) adaptor protein to the damage site and activation of Rad53 DDC effector kinase (CHK2 in humans), leading to cell cycle arrest and induction of transcription of DNA repair factors [30]. In yeast, virtually all DSBs undergo resection, which is a process of strictly controlled enzymatic degradation of the 5′ end of broken DNA. In yeast, as well as in mammals, resection is initiated by the Mre11-Sae2 complex (CtIP in humans) and is further catalyzed by the Exo1 exonuclease (EXO1 in humans) or the Dna2 nuclease (DNA2 in humans) in a complex with the Sgs1 helicase (BLM or WRN in humans) [31]. Single-stranded DNA (ssDNA) generated as a result of DNA resection is immediately coated by a replication protein A (RPA) complex preventing unscheduled DNA degradation and formation of secondary DNA structures. Moreover, RPA is a binding platform for a Ddc2 protein (ATRIP in humans) that recruits the second, essential DDC sensor kinase Mec1 (ATR in humans). Like Tel1, Mec1 phosphorylates histone H2A on serine 129, allowing Rad9 recruitment and full activation of Rad53 effector kinase. Moreover, Mec1 plays a crucial role in replication fork stabilization during genotoxic stress conditions [30]. 

In response to different types of DNA damage, specific repair pathways are activated. While chemically modified DNA bases (e.g., oxidized or methylated) are repaired by base excision repair (BER) [32], bulky adducts (e.g., DNA crosslinks or pyrimidine dimers) are removed by nucleotide excision repair (NER) [33]. On the other hand, stalled replication forks as well as DNA breaks are mainly repaired by homologous recombination (HR) with a minor role of nonhomologous recombination (NHEJ) in yeast [34,35]. HR is prevalent in the S phase and G2 phase of the cell cycle as it is dependent on Cdc28 activity, which is inhibited in G1 phase [36]. HR is a complex process that can be performed in a Rad51-dependent and independent manner [37,38]. In the first case, Rad52 (BRCA2 in humans) mediates the exchange of RPA molecules for Rad51. This enables formation of a nucleofilament structure that allows the use of homologous DNA as a template to repair broken DNA [39]. Alternatively, if DSB is created between two repeated sequences oriented in the same direction, complementary, single-stranded sequences generated by resection can be annealed in a process that depends on Rad52 and Rad59 [40].

It has been shown that the accumulation of reactive oxygen species (ROS) results in the appearance of oxidative stress. Numerous studies indicate that DNA repair mechanisms (mainly BERs) are activated in response to oxidative DNA damage (oxidized DNA bases, DNA single- and double-strand breaks) [41,42,43,44,45]. In human cells it was demonstrated that the presence of hydrogen peroxide and tertiary-butyl hydro peroxide causes ROS accumulation leading to oxidative stress which results in induction of DNA damage also in the form of DNA breaks [46]. Furthermore, in yeast cells, exposure to DNA oxidizing agents may cause single- as well as double-breaks of DNA [47]. Previous studies were focused on the influence of 3-BP on cell metabolism, identification of the pathways of 3-BP uptake and extrusion and potential resistance mechanisms [3]. Here we investigate whether 3-BP is also a genotoxic agent under the conditions where most cells are still viable. Our results indicate that in yeast, as well as in human cancer cells, 3-BP induces DNA damage mostly in the form of DSBs probably as a result of generation of high levels of ROS. The significance of our discovery for potential use of 3-BP in anticancer therapy is discussed.

## 2. Materials and Methods

### 2.1. Yeast Strains and Growing Conditions

The yeast strains used in this study were isogenic derivatives of W303 and are listed in Table 1. Yeast cells were cultured in liquid SD minimal medium (0.67% yeast nitrogen base, 2% sucrose, 5 mg/mL adenine, 2 mg/mL histidine, 6 mg/mL leucine, 4 mg/mL tryptophan, 2 mg/mL uracil) at 30 °C. To solidify SD medium, 2% agar was added. 

### 2.2. Yeast Sensitivity Tests 

To determine the sensitivity of relevant yeast strains to 3-BP, logarithmically growing cell cultures were 10-fold serially diluted and spotted on solid media containing the indicated concentrations of 3-BP. To assess the survival rate of the wild type strain after acute 3-BP treatment, cells were exposed to various concentrations of the compound for indicated periods of time or left untreated. Next, cells were extensively washed and diluted before plating on solid SD medium.

### 2.3. Cell Cycle Synchronization

To arrest yeast in G1 phase, cells were incubated with 5 µM α-factor for 2 h. To synchronize yeast at the G2/M boundary, cells were treated with 15 µg/mL of nocodazole for 2 h. Proper cell cycle synchronization was confirmed by microscopy observations and fluorescence-activated cell sorting (FACS) analysis. 

### 2.4. Live Cell Imaging

Cells expressing Mre11-YFP, Rfa1-YFP and Rad52-YFP were observed with the Axio Imager M1 epifluorescence microscope (Carl Zeiss, Göttingen, Germany) equipped with a 100× immersion oil objective (Plan-Neofluar 1006/1.30), with the GFP filter set at differential interference contrast (DIC). Images were collected using an AxioCam MRc digital color camera and processed with AxioVision 4.5 software.

### 2.5. Cell Cycle Analysis

To analyze the cell cycle progression of yeast cells, at indicated time points 0.5 mL of yeast cultures were fixed with 70% ethanol and then extensively washed with water. Next, cells were incubated for 2 h with 0.25 µg/mL RNase at 50 °C followed by 1 h incubation with 1 µg/mL pepsin at 37 °C. After several washes with water, cells were sonicated, stained with 2.5 µM SYTOX Green for 30 min and then analyzed by flow cytometry using Guava^®^ easyCyte (Millipore, Molsheim, France). To determine the fraction of cells remaining arrested in G1 we performed an α-factor-nocodazole trap assay. At every time point, 0.5 mL of yeast culture was washed with water and transferred to 0.5 mL of SD medium containing 5 µg/mL α-factor and 15 µg/mL nocodazole. Cell arrest in G1 is sustained by α-factor while cells that enter S phase are unresponsive to α-factor and accumulate in G2/M. After 90 min of incubation at 30 °C cells were fixed with 70% ethanol and processed for flow cytometry analysis. To assess the fraction of post-mitotic cells, ethanol-fixed cells were processed as for flow cytometry, and then observed with an Axio Imager M1 epifluorescence microscope (Carl Zeiss, Göttingen, Germany) to score the percentage of binucleate large-budded cells. All cell cycle experiments were repeated a minimum of three times.

### 2.6. ROS Measurements

To measure the levels of reactive oxygen species in yeast, wild type cells were pre-loaded with 5 µg/mL dihydrorhodamine 123 or dihydroethidium for 15 min and then exposed to various concentrations of 3-BP. At indicated time points aliquots of cells were taken and immediately analyzed with an epifluorescence microscope to estimate the levels of green and red fluorescence of rhodamine 123 and 2-hydroxyethidium, respectively, formed after oxidation of the probes. Untreated samples were used as a control of autofluorescence level. Fluorescence was detected with an Axio Imager M1 epifluorescence microscope (Carl Zeiss, Göttingen, Germany) or with a Varioskan LUX multimode microplate reader (ThermoFisher Scientific, Waltham, MA, USA).

### 2.7. Yeast Neutral Comet Assay

For yeast neutral comet assay 10^5^ cells were collected, centrifuged and washed with S-buffer (1 M sorbitol, 25 mM KH_2_PO_4_, pH 6.5). Next, cells were resuspended in 1.5% low melting agarose dissolved in S buffer containing 2 mg/mL Zymolyase 20T. Subsequently, 40 µl of cell suspension was spread on microscope slides, overlaid with 1% agarose and covered with coverslips followed by 20 min incubation at 37 °C. Next, slides were incubated for 5 min at 4 °C and the coverslips were removed. In the next step slides were incubated in cold lysis solution (150 mM NaCl, 30 mM EDTA, 10 mM Tris-HCl, 0.1% N-lauroyl sarcosine, pH 8.3) for 20 min and washed in TBE buffer, pH 8.3 for 5 min. DNA electrophoresis was conducted at 4 °C for 15 min at 0.45 V/cm in TBE buffer, pH 8.3. After electrophoresis slides were soaked for 5 min in water, followed by sequential incubation in 75% and 95% ethanol for 5 min. Finally, slides were left to air dry for 15 min, stained with SYTOX Green and observed with a Carl Zeiss Axio Imager M1 epifluorescence microscope. Comet tail lengths were analyzed using CometScore 2.0 software. At least 50 comets were analyzed per slide.

### 2.8. Pulsed-Field Gel Electrophoresis

Pulsed-field gel electrophoresis of yeast cells was performed as described by Litwin et al. [52]. Briefly, 3 × 10^7^ cells were embedded in low melting agarose and digested with lyticase for 2 h at 37 °C (10 mM Tris, pH 7.2, 50 mM EDTA, 1 mg/mL lyticase). Next, cells were washed and incubated overnight with proteinase K at 50 °C (100 mM EDTA, pH 8.0, 0.2% sodium deoxycholate, 1% sodium lauryl sarcosine, 1 mg/mL proteinase K). Yeast chromosomes were resolved in 1% agarose at 6 V/cm for 24 h with a 60–120s switch time ramp and 120° switch angle at 4 °C using a CHEF-DR III pulsed-field electrophoresis system (Bio-Rad, Hercules, CA, USA). For pulsed-field electrophoresis of human DNA, 10^5^ cells were embedded in low melting agarose and incubated with proteinase K for 24 h (0.5 M EDTA, 1% N-laurylsarcosyl, proteinase K 1 mg/mL). Next, agarose plugs were washed with TE buffer and the DNA was resolved in 1% agarose at 4 V/cm for 24 h with a 60–240 s switch time ramp and 120° switch angle at 14 °C. To visualize the DNA electrophoresis, gels were stained with ethidium bromide and analyzed using Bio-Rad ChemiDoc MP System and Image Lab software (Software 6.0.1, Hercules, CA, USA).

### 2.9. Protein Analysis

Total protein extracts were extracted by the TCA method. The cell pellet was resuspended in 2 M NaOH, 7% β-mercaptoethanol, followed by addition of 50% trichloroacetic acid. After centrifugation the precipitate was washed with 1 M Tris pH 8.0 and centrifuged. Proteins were resuspended in Laemmli buffer (62.5 mM Tris pH 6.8, 20% glycerol, 2% SDS, 5% β- mercaptoethanol, 0.15% bromophenol blue). Proteins were resolved on 12% SDS–PAGE gel, blotted onto nitrocellulose membranes and then probed with anti-H2A P-S129 (Abcam, ab17353). To confirm equal protein loading, membranes were stained with Ponceau S before immunodetection.

### 2.10. Mammalian Cell Culture

THP-1 cell line (ATCC, TIB-202, Manassas, VA, USA) was maintained at a cell density of 2 × 10^5^–8 × 10^5^ mL in RPMI 1640 medium (Lonza, Bornem, Belgium) supplemented with 10% heat inactivated fetal bovine serum (GIBCO, Life Technologies, Waltham, MA, USA), 1× antibiotic-antimycotic solution (GIBCO, Life Technologies), 1 × glutamax (GIBCO, Life Technologies) in humidified 5% CO_2_ atmosphere at 37 °C. To determine cell viability, cells were grown up to 24 h in fresh complete medium at a concentration of 1 × 10^6^ cells/mL in the presence of various concentrations of 3-BP. The number of viable cells was determined by a standard trypan blue exclusion assay using a 0.4% Trypan blue solution (w/v) and a Thoma hemocytometer. The experiment was repeated three times using the THP-1 line from passages 4, 5 and 8. For each experiment the control and 3-BP treated samples were prepared simultaneously in two repetitions. At time 0 and after 12 h and 24 h of incubation two experts performed a double-blind evaluation of the viability of a set of 2 single-cell suspensions, making a total of 12 sample counts for each option (4 counts per experiment). The cell viability was determined in reference to the initial control sample treated as 100%.

### 2.11. Mammalian Alkaline Comet Assay 

THP-1 cells were treated with different 3-BP concentrations and incubated for 12 h. Next, cells were washed with PBS (lacking divalent cations) and cell density was adjusted to 2 × 10^4^ cells/mL. Then, 0.4 mL of cell suspension was mixed with 1.2 mL of solution of 1% low-gelling-temperature agarose in PBS (maintained at 37 °C) and spread onto a microscope slide precoated with 1% agarose. Slides were incubated for 2 min at 4 °C and treated with lysis solution (1.2 M NaCl, 0.26 M NaOH, 100 mM Na_2_EDTA, 0.1% sodium lauryl sarcosinate, pH > 13) overnight at 4 °C. The next day slides were washed 3 × 15 min in electrophoresis solution (0.03 M NaOH, 2 mM Na_2_EDTA, pH~12.3) and then submerged in electrophoresis solution in an electrophoresis chamber. Electrophoresis was conducted for 25 min at 0.6 V/cm. Slides were removed from the electrophoresis chamber and neutralized in water. DNA was stained with 5 µM SYTOX Green solution for 5 min and analyzed with an epifluorescence microscope. Comets were analyzed using OpenComet v1.3.1 software. At least 50 comets were analyzed per slide. 

### 2.12. H2A.X Phosphorylation Assay

The percentage of H2A.X activated cells was measured using the Muse Multi-Color DNA Damage Kit and Muse Cell Analyzer according to the manufacturer’s instructions. 

## 3. Results

### 3.1. Replication-Dependent Activation of DNA Damage Checkpoint by 3-BP in Yeast

**Figure 1:**
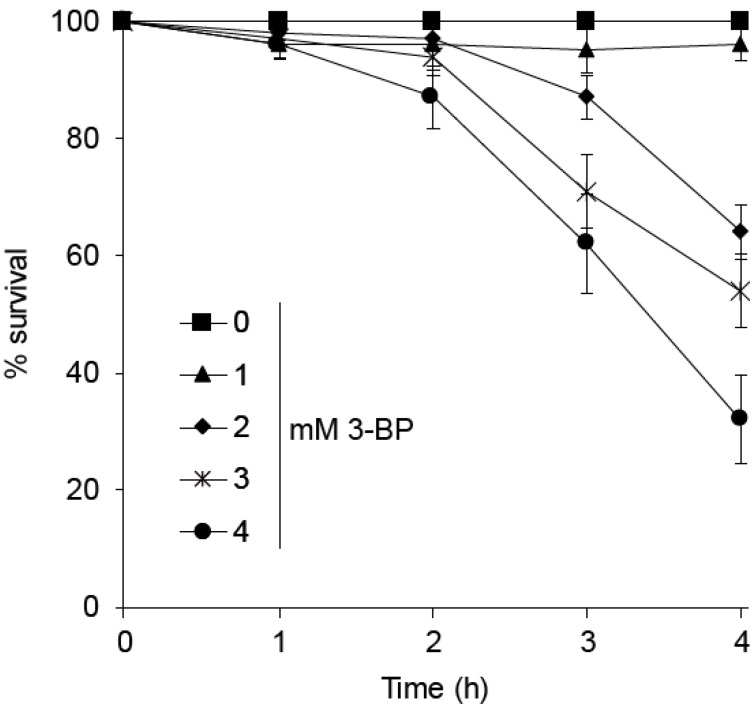
Wild type sensitivity to acute 3-bromopyruvate (3-BP) treatment. Logarithmically growing wild type cells were treated with various 3-BP concentrations for indicated time points. Next, cells were washed and plated on solid SD medium. Survival was calculated as percentage of colony forming units of 3-BP treated cells versus untreated cells. Error bars represent mean value ± standard deviations of mean (*n* = 3).

To assess the impact of 3-BP on wild type (WT) cell viability we performed a survival assay. Yeast cells were incubated for indicated time points with various concentrations of the drug, then washed and plated on solid medium. Since we have previously shown that in yeast 3-BP is taken up by the Jen1 symporter, which is repressed in the presence of glucose, we cultured all yeast strains on minimal medium containing sucrose to allow JEN1 expression and 3-BP uptake. Incubation of wild type cells for 1 h in the presence of up to 4 mM of 3-BP did not lead to any viability loss (Figure 1). Furthermore, two-hour exposure to the drug was not toxic to the cells. After 3 h of incubation about 30% of the cells lost viability when exposed to 3 mM of 3-BP and 40% when treated with 4 mM of the drug. However, a 4-h exposure of 3-BP at lower concentrations of 2 mM to 4 mM led to reduced cell survival (Figure 1). Taken together, these results show that 3 h of incubation with up to 3 mM 3-BP is not toxic for the cells.

**Figure 2:**
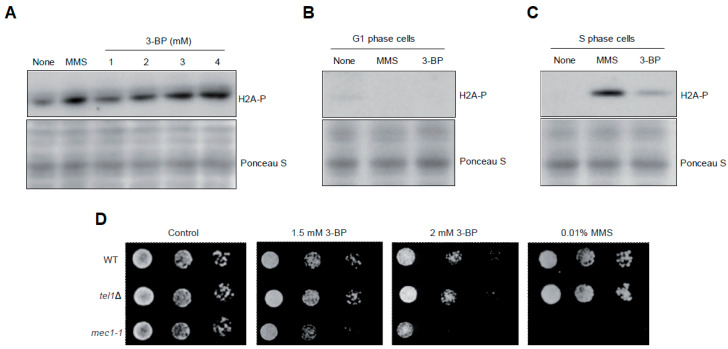
Cell cycle phase-dependent activation of DNA damage checkpoint by 3-BP in budding yeast: (**A**) Exposure to 3-BP leads to histone H2A phosphorylation. The logarithmically growing culture of wild type cells was treated with increased concentrations of 3-BP for 2 h and then total proteins were isolated. Next, protein extracts were analyzed with Western blot using anti-phosphoS129 H2A antibodies. To confirm equal protein loading, membranes were stained with Ponceau S. Methyl methanesulfonate (MMS) was used as an agent with known DNA-damaging activity. (**B**) Cells treated with 3-BP in G1 phase do not induce checkpoint. Cells were arrested in G1 phase with α-factor and then treated with 3 mM 3-BP for 2 h in the presence of alpha-factor. Next, total proteins were isolated and analyzed with Western blot using anti-phosphoS129 H2A antibodies. (**C**) 3-BP triggers DNA damage checkpoint activation during the S phase of the cell cycle. Cells were arrested in the G1 phase with α-factor and then washed and released to fresh medium. After 20 min cells were exposed to 3 mM 3-BP for 2 h. Next, total proteins were isolated and subjected to Western blot analysis using anti-phosphoS129 H2A antibodies. (**D**) A mec1-1 DNA damage checkpoint mutant shows increased sensitivity to 3-BP. Serial dilutions of indicated strains were plated on solid media in the presence or absence of 3-BP at 30 °C and photographed after 3 days.

To determine whether 3-BP generates DNA damage we decided to follow induction of the DNA damage checkpoint by monitoring the levels of histone H2A (yeast H2AX) phosphorylation at S129 (H2A-P), which is considered to be a sensitive marker of both DSBs and replication fork stalling [53,54]. As shown before, untreated, logarithmically growing wild type cells exhibit low levels of spontaneous H2A phosphorylation while treatment with methyl methanesulfonate (MMS), which methylates DNA bases leading to fork stalling, causes strong induction of H2A-P. Interestingly, exposure to 3-BP led to a dose-dependent increase of histone H2A phosphorylation (Figure 2A). Next, we examined whether induction of H2A phosphorylation depends on DNA replication. To test this, wild type cells were synchronized in G1 with α-factor and treated with 3 mM 3-BP or 0.05% MMS for 2 h. Alternatively, G1-synchronized cells were released in the absence of α-factor to allow progression into S phase and after 20 min were exposed to 3 mM 3-BP or 0.05% MMS for 2 h. We found that, like MMS, 3-BP is unable to induce a DNA damage checkpoint outside of S phase (Figure 2B) but clearly triggers H2A phosphorylation during DNA replication (Figure 2C). Finally, to assess the importance of the DNA damage checkpoint for cell viability in the presence of 3-BP we performed spot assays using strains lacking Mec1 and Tel1 sensor kinases. We found that cells devoid of MEC1, but not TEL1, were more sensitive to 3-BP than WT cells, suggesting an important role for DNA damage checkpoint activation in coping with 3-BP toxicity (Figure 2D). Taken together, these results suggest that 3-BP induces replication-dependent DNA damage.

### 3.2. 3-BP Triggers Cell Cycle Delay In All Cell Cycle Phases

**Figure 3:**
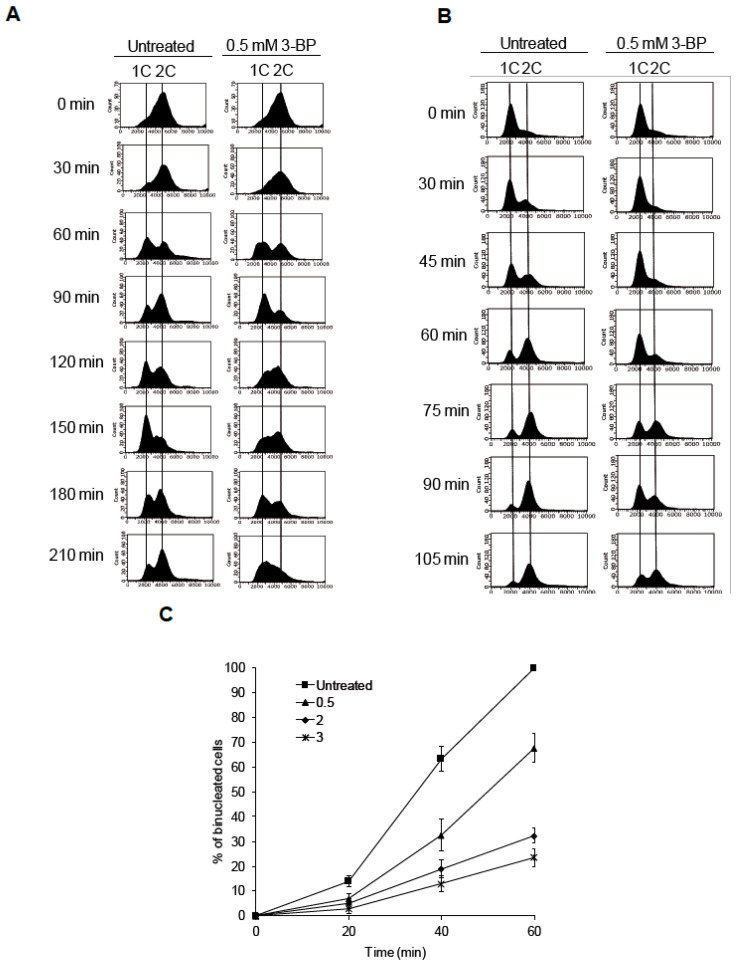
Cell cycle analysis of cells treated with 3-BP: (**A**) Cells exposed to 3-BP show slower progression through S phase of the cell cycle. Wild type cells were synchronized in G2 phase with nocodazole then washed and resuspended in fresh medium in the presence or absence of 3-BP. At indicated time points cells were collected and processed for flow cytometry analysis. (**B**) Cells treated with 3-BP accumulate at the G1/S boundary. To determine the fraction of cells remaining in the G1 cell cycle phase after 3-BP exposure, an α-factor-nocodazole trap assay was performed. Cell cycle distribution was assessed by flow cytometry. (**C**) Presence of 3-BP delays cell progression into mitosis. Wild type cells were synchronized in G2 phase with nocodazole then washed and resuspended in fresh medium in the presence or absence of 3-BP. At indicated time points cells were collected and stained. The percentage of bi-nucleate cells was counted using fluorescent microscopy.

To evaluate the impact of 3-BP on cell cycle progression we performed FACS analysis of wild type cells synchronized in G2/M and then released to fresh medium containing or not containing the drug. In the untreated culture, wild type cells started to accumulate in G1 60 min after release and 30 min later most cells reached G2/M phase again, continuing the cell cycle (Figure 3A). Interestingly, cells treated with 3-BP also reached G1 after 60 min, but in contrast to untreated culture remained in this phase for another 30 min. A total of 120 min after release to 3-BP-containing media, cells show clear accumulation in S phase and slow progression through the cell cycle at the next time points (Figure 3A). Next, we analyzed the dynamics of G1/S transition in the presence of 3-BP by α-factor/nocodazole trap assay. It was found that cells treated with the drug remain in G1 for a longer time than untreated cells (Figure 3B). Finally, we assessed the G2/M arrest duration in WT cells after 3-BP treatment. We found that in the presence of 3-BP, cells delay progression to mitosis (Figure 3C). Taken together, these data suggest that in response to 3-BP cells slow down cell cycle progression, accumulating in all cell cycle phases.

### 3.3. Exposure to 3-BP Leads to Generation of Reactive Oxygen Species in Yeast

**Figure 4:**
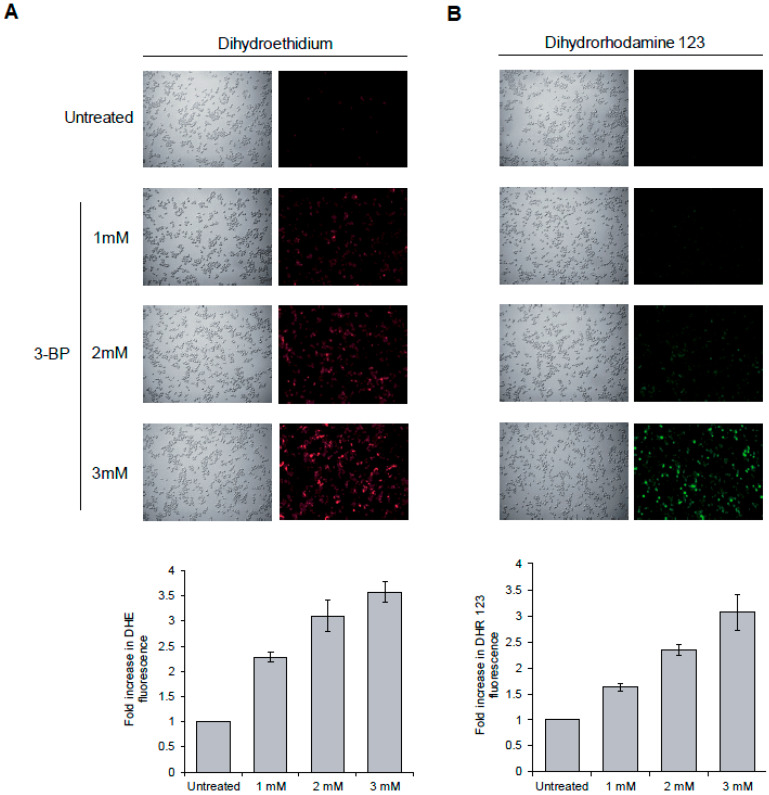
3-BP induces reactive oxygen species (ROS) formation. (**A**, **B**) 3-BP0000000 treatment results in generation of ROS. A logarithmically growing culture of wild type cells pre-loaded with dihydroethidium (**A**) or dihydrorhodamine 123 (**B**) was treated with indicated concentrations of 3-BP for 2 h. Fluorescence was detected using a fluorescence microscope and quantified with a fluorescence plate reader. Error bars represent mean value ± standard deviations of mean (*n* = 3). Average fluorescence value for untreated cultures was set to 1.

It was recently revealed that in human cancer cells 3-BP triggers formation of ROS [14,15,16,17]. Since oxidative DNA damage may induce checkpoint activation in S phase [55], we decided to determine whether in yeast cells 3-BP also causes oxidative stress. To test this, we incubated wild type cells with two ROS probes, dihydrorhodamine 123 and dihydroethidium, that convert to fluorescent products when oxidized. While untreated cells showed low levels of fluorescence, 2 h of incubation with up to 3 mM of 3 BP caused a strong increase of fluorescence levels of both compounds used as ROS probes (Figure 4A,B). These results show that, as in mammalian cells, in yeast 3-BP also induces formation of ROS, suggesting that 3-BP may cause oxidative DNA damage.

### 3.4. The role of DNA Repair Pathways in the Tolerance to 3-BP in Yeast

**Figure 5:**
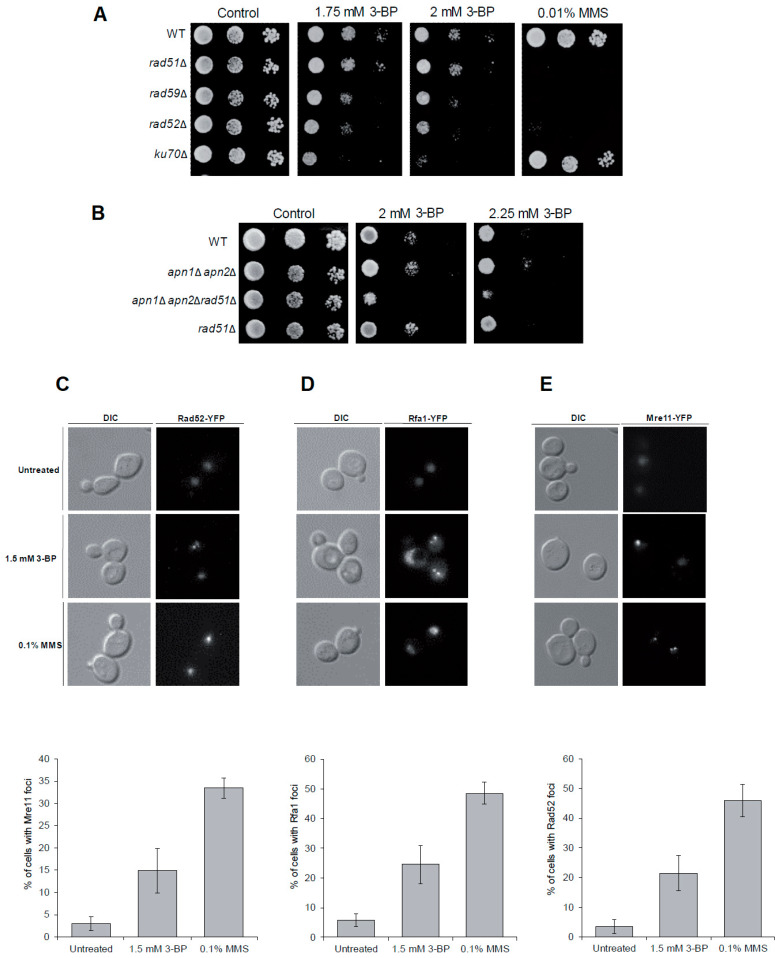
Homologous recombination is crucial for tolerance to 3-BP: (**A,B**) Homologous recombination, nonhomologous end-joining and base excision repair pathways are required for maintaining viability of yeast cells in the presence of 3-BP. Serial dilutions of indicated strains were plated on solid media in the presence or absence of 3-BP at 30 °C and photographed after 3 days. (**C**–**E**) 3-BP treatment triggers formation of homologous recombination repair centers. Logarithmically growing cultures of cells bearing Mre11-YFP (**A**), Rfa1-YFP (**B**) and Rad52-YFP (**C**) were treated with 3-BP or MMS for 1.5 h. Next, cells were washed and processed for microscopic analysis. Error bars represent mean value ± standard deviations of mean (*n* = 3).

To test our hypothesis that 3-BP is a genotoxic agent we investigated which DNA repair pathway protects yeast against 3-BP by comparing growth of wild type and mutant cells devoid of the BER (*apn1*Δ*apn2*Δ), NHEJ (yku70Δ) and HR pathway (*rad51*Δ, *rad59*Δ, *rad52*Δ) in the presence of 3-BP. We found that single mutants lacking NHEJ or genes encoding proteins crucial for Rad51-independent HR (*RAD52* and *RAD59*) were important for viability in the presence of 3-BP (Figure 5A). Next, we constructed yeast strains lacking BER and Rad51-dependent HR. Interestingly, although *apn1*Δ*apn2*Δ and r*ad51*Δ mutants grew as WT, the triple mutant showed strong sensitivity to the drug (Figure 5B). Finally, to show that DNA damage induced by 3-BP is actively repaired by HR we monitored the levels of Mre11-YFP, part of the DSB sensor complex MRX, Rfa1-YFP, a large subunit of the RPA complex, and the HR recombinase Rad52-YFP. All these proteins accumulate at damage sites creating distinct fluorescence foci representing DNA repair centers. [56] We found that logarithmically growing cells exposed to 1.5 mM 3-BP for 1.5 h show a 4–5-fold increase in the number of cells with Mre11, Rfa1 and Rad52 foci over spontaneous levels (Figure 5C–E). Taken together, these data suggest that 3-BP induces mostly DNA breaks repaired by HR, and to a lesser degree by NHEJ, as well as some oxidative DNA damage repaired by BER.

### 3.5. 3-BP Induces DNA Double-Strand Breaks in Yeast

**Figure 6:**
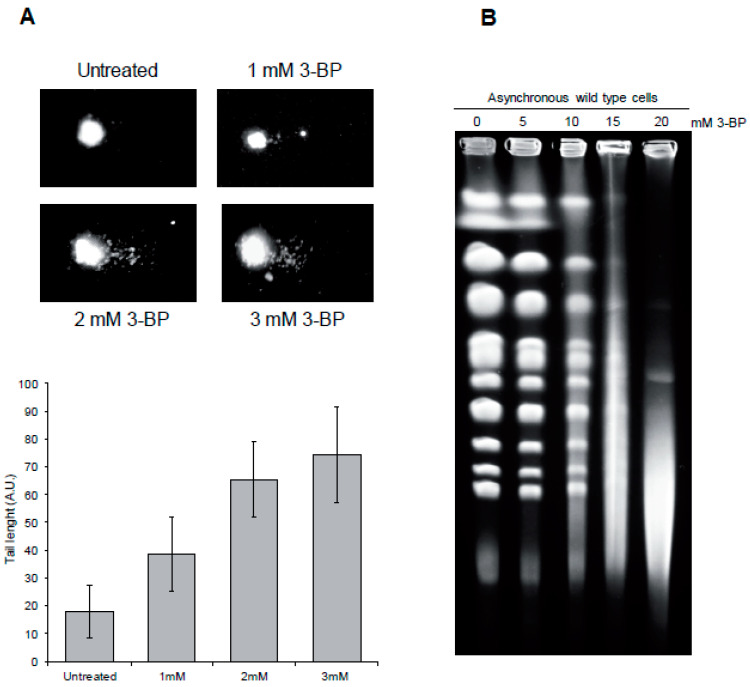
3-BP treatment leads to DNA double-strand break formation in yeast: (**A**) 3-BP induces DNA breakage in yeast as revealed by the comet assay. Asynchronous wild type cells were exposed to indicated 3-BP concentrations for 2 h or left untreated followed by a single-cell gel electrophoresis. Representative images of comets are shown. Error bars represent mean value ± standard deviations of mean (*n* = 3). (**B**) Exposure to 3-BP results in generation of DNA double-strand breaks. A logarithmically growing culture of wild type cells was treated with indicated concentrations of 3-BP for 5 h. Next, intact chromosomes were isolated and processed for pulsed-field gel electrophoresis (PFGE).

To demonstrate the presence of DNA breaks generated by 3-BP directly, we performed a neutral comet assay, which detects mostly DNA double-strand breaks. In this method, DNA isolated from individual yeast cells is subjected to electrophoresis which allows slow-migrating, undamaged DNA (comet head) to be separated from faster, broken DNA (comet tail). Interestingly, exposure to 3-BP resulted in more DNA migrating in the tail, suggesting that this compound induces DNA breaks, most likely in the form of DNA double-strand breaks (Figure 6A). To confirm the presence of DSBs in WT cells after exposure to 3-BP we performed pulsed-field gel electrophoresis (PFGE) of budding yeast chromosomes isolated from asynchronous cultures treated with 3-BP. The presence of DSBs can be visualized as the disappearance of individual chromosome bands and accumulation of a low molecular weight smear. As PFGE is a specific but not sensitive method of DSB detection, we exposed yeast cells to much higher concentrations of 3-BP. It was found that exposure to 3-BP triggers chromosome fragmentation and appearance of a low molecular weight smear (Figure 6B). Taken together, these data suggest that 3-BP induces formation of DNA double-strand breaks.

### 3.6. 3-BP Causes DNA Damage Checkpoint Activation and Induction of DNA Double-Strand Breaks in Mammalian Cells

**Figure 7:**
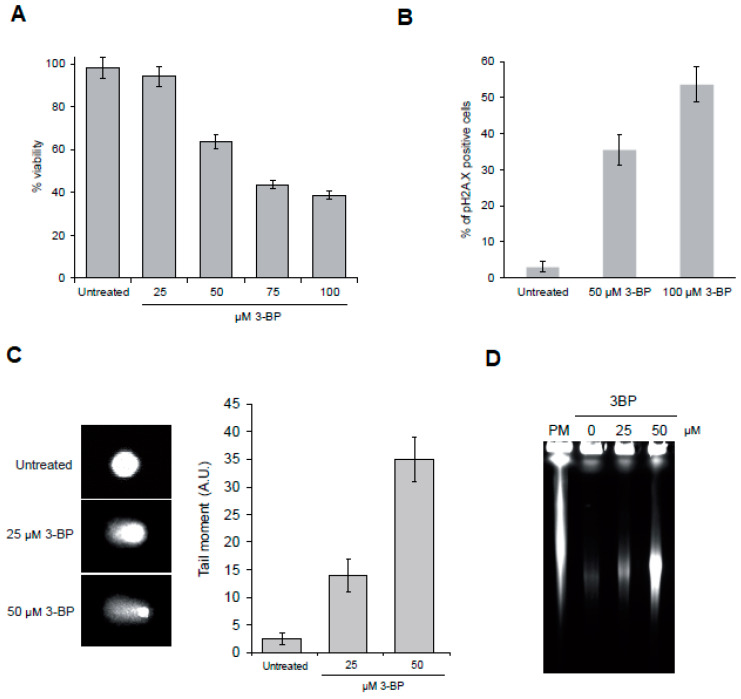
3-BP induces DNA double-strand break formation in mammalian cells: (**A**) 3-BP decreases the viability of THP-1 cell line. THP-1 cells were treated with different 3-BP concentrations for 12 h and cell viability was assessed by trypan blue exclusion test. Error bars represent mean value ± standard deviations of mean (*n* = 3). (**B**) 3-BP triggers DNA damage checkpoint activation in mammalian cells. Cells were treated with indicated concentrations of 3-BP for 12 h and processed for analysis of cells with phosphorylated histone H2A.X. (**C**) 3-BP induces formation of DNA breaks in THP-1 cells. THP-1 cells were treated with indicated concentrations of 3-BP for 12 h and then processed for alkaline comet assay. Representative images of comets are shown. Error bars represent mean value ± standard deviations of mean (*n* = 3). (**D**) Exposure to 3-BP results in generation of DNA double-strand breaks. THP-1 cells were treated with indicated concentrations of 3-BP for 12 h. Next, intact chromosomes were isolated and processed for PFGE.

Taking into account our results in yeast we wondered whether 3-BP also induces DNA damage in human cancer cells. First, we assessed the impact of 3-BP on the THP-1 cell line viability. We found that incubation with up to 25 µM of 3-BP has no impact on cell viability. On the other hand, treatment with 50 µM of 3-BP leads to approximately 40% viability loss. Higher concentrations of the drug cause even higher cell mortality (Figure 7A). To determine whether 3-BP can trigger DNA damage we assessed the induction of H2A.X phosphorylation after 3-BP treatment [57,58,59]. We found that only 3% of untreated cells displayed the H2A.X signal. On the other hand, exposure to 50 and 100 µM of 3-BP led to H2A.X signal appearance in 35% and 53% of cells, respectively (Figure 7B). Next, we performed the alkaline comet assay to investigate the presence of DNA breaks. We found that 3-BP treatment leads to increased migration of the DNA in the comet tail, indicating DNA breakage (Figure 7C). Finally, to determine whether 3-BP induces DSBs we performed PFGE. In contrast to yeast chromosomes, human chromosomes are too large to be separated during electrophoresis and only smaller, broken DNA fragments can enter the gel, migrating as a smear. As a positive control for a DSB inducer we used the radiomimetic drug phleomycin. Almost no fragmentation of DNA was found in samples isolated from untreated cultures while DNA from cells exposed to PM was heavily broken. Importantly, exposure to 3-BP also led to DNA fragmentation, suggesting the presence of DSBs (Figure 7D). Taken together, these data indicate that, as in yeast, 3-BP generates DNA breaks.

## 4. Discussion

In the era of aging societies, every year more and more people are suffering from cancer. Because standard treatments are mostly toxic not only for malignant cells but also for normal tissues, the introduction of new anticancer agents is extremely important. 3-bromopyruvate (3-BP) is an intensively studied compound exhibiting anticancer and antimicrobial activities [1,2,3]. Interestingly, 3-BP shows strong selectivity against many cancers. It was demonstrated that it accumulates mostly in malignant cells, which overexpress mono-carboxylate transporters to export lactic acid, while being relatively nontoxic for healthy cells which produce little lactate [4,5] under normal physiological conditions.

It has been shown that the main mechanism of 3-BP cytotoxicity is based on its ability to disturb biological systems responsible for energy production, thus rapidly depleting ATP levels. Indeed, it was reported that 3-BP inhibits several glycolytic enzymes including hexokinase II, glyceraldehyde 3-phosphate dehydrogenase and lactate dehydrogenase [7,8,9,10,11,12]. This is especially deleterious for cancers that exhibit the Warburg effect, namely enhanced glycolysis even in the presence of oxygen. Moreover, it was shown that 3-BP affects the respiratory chain by inhibiting complexes I and II, further leading to ATP depletion [13]. 

In this study we discovered that 3-BP treatment not only blocks energy production but also leads to induction of DNA damage both in the yeast Saccharomyces cerevisiae and in human cancer cells. First, exposure to 3-BP activates the DNA damage checkpoint pathway, leading to phosphorylation of H2A histone (H2A.X in human), a sensitive marker of both single- (SSBs) and double-strand breaks (Figure 2A and Figure 7B). Secondly, we were able to directly show the presence of DNA breaks, in both types of cells treated with 3-BP, by a comet assay (Figure 6A and Figure 7C). Finally, using PFGE, we specifically demonstrated the presence of DNA double-strand breaks in yeast and human cancer cells (Figure 6B and Figure 7D). These data are in good agreement with a recent report showing that 24 h exposure to 10 µM 3-BP increases the levels of 53BP1-GFP foci in human cancer cells, suggesting induction of DNA damage [60]. On the other hand, in contrast to our results, a previous report revealed no induction of H2A.X phosphorylation and ATM activation in human cell lines after 4 h treatment with 300 µM of 3-BP [61]. Why these results differ is not entirely clear, but it may involve the use of distinct cell lines, different methods and/or exposure conditions.

Using yeast as a model organism we also identified pathways that allow repair of DNA damage induced by 3-BP. Both HR and NHEJ were found to be important for resistance to 3-BP (Figure 5A). Moreover, we were able to observe the formation of Mre11-YFP, Rfa1-YFP and Rad52-YFP foci that represent repair centers where DNA damage is actively repaired by HR (Figure 5C–E). Interestingly, our analysis of multiple mutants revealed that lack of BER also renders cells sensitive to 3-BP (Figure 5B). Taken together, these data further suggest that 3-BP mostly induces DNA breaks (SSBs and DSBs) that are repaired mainly by HR and NHEJ, but it also induces some damage of DNA repaired by BER.

Importantly, we revealed that in yeast 3-BP triggers H2A phosphorylation only in S phase synchronized cells as no DDC activation was detected in G1-arrested cells (Figure 2B). These data imply that generation of DNA breaks by 3-BP requires either the presence of unwound dsDNA and/or an active process of DNA replication. In support of these results we found that exposure to 3-BP leads to slower progression through the S phase, probably as a result of DDC activation (Figure 3A). This is supported by the results showing that cells lacking the main DDC kinase Mec1 kinase were sensitive to 3-BP (Figure 2D). It is worth noting that 3-BP treatment slows down G1 to S phase transition and progression through mitosis despite the lack of DDC activation in G1 phase (Figure 3B,C). In the presence of 3-BP, all phases of the cell cycle are slowed and extended (Figure 3A,B). After 60 min, untreated cells continue the cell cycle while 3-BP treated cells are still, in the vast majority, in G1 phase (Figure 3B). This delay of cell cycle kinetics is also visible after the release of cells synchronized with nocodazole in the G2/M phase (Figure 3A). The observed cell cycle slowdown in the presence of 3-BP is probably associated with disorders leaving the G1 phase or passing the S phase. Another result indicates problems at the G2/M boundary (Figure 3C) and also a delay of cell cycle kinetics upon 3-BP treatment. Interestingly, despite the fact that cell cycle slowdown in the presence of 3-BP is observed in all phases, H2A phosphorylation is only observed in the S phase. This may be due to damage during replication and the presence of replication stress. How 3-BP may induce cell cycle delay in all phases of the cell cycle is not clear. It has been demonstrated that 3-BP forms complexes with glutathione, leading to a strong decrease in the intracellular glutathione levels both in human cancer and in fungal or algal cells [19,20,21]. Interestingly, low levels of glutathione lead to prolonged cell cycle arrest [62].

As glucose-6-phosphate formation is inhibited by 3-BP due to hexokinase inhibition, 3-BP can lead to inhibition of the pentose phosphate pathway (PPP). This, in turn, can cause a fall in nicotinamide adenine dinucleotide phosphate hydrogen (NADPH) levels, a further decrease in glutathione (GSH) content and an increase in ROS levels. In addition, PPP is a major source of nucleoside triphosphate (dNTPs), and action of 3-BP could lead to a decrease in the dNTP pool. As dNTPs are crucial for DNA repair and cell survival in the presence of DNA damage [63,64], DNA damage induced by 3-BP could be exacerbated by smaller dNTP pools. This mechanism could make DNA-damaging chemotherapeutics more effective and act in a synergistic manner with 3-BP.

It is not entirely clear how 3-BP induces DNA breaks. 3-BP treatment probably leads to generation of ROS through its cellular metabolism, by damaging mitochondria and as a result of GSH depletion [20,26,27]. Recent studies also show that 3-BP may directly disrupt the OXPHOS electron transport chain in mitochondria and thereby induce ROS [65]. It is also known that at least some chemical oxidants such as H_2_O_2_ or bleomycin are able to induce both SSBs and DSBs [66,67,68]. Here we have shown that also in budding yeast exposure to 3-BP results in elevated levels of ROS leading to oxidative stress (Figure 4A,B). As accumulation of DSB correlates with increased ROS levels induced during 3-BP treatment, these data suggest that the DNA breaks formed in 3-BP-treated cells may be a result of oxidative DNA damage and possibly not through direct 3-BP action.

**Figure 8:**
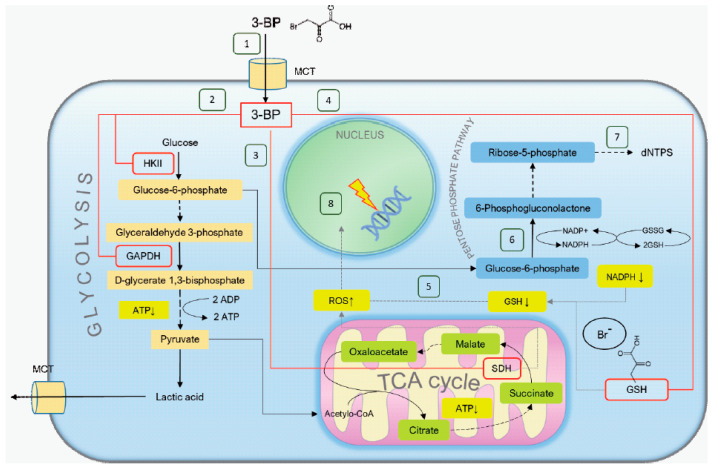
Proposed 3-bromopyruvate mechanism of action: (1) 3-bromopyruvate enters cells through MCTs (mono-carboxylate transporters), which are overexpressed in many cancer cells. (2) Following 3-BP entrance the molecule reacts with cysteine residues of proteins, leading to loss of tertiary structure and function [73]. 3-BP reacts with glycolysis enzymes hexokinase 2 (HKII) and glyceraldehyde 3-phosphate dehydrogenase (GAPDH), leading to depletion of ATP. Moreover, 3-BP inhibits succinate dehydrogenase (SDH), respiratory complex II, which oxidizes succinate to fumarate (3) [74], which can lead to disruption of ATP synthesis and the electron transport chain causing electron leakage and generation of reactive oxygen species (ROS) (4) [75,76]. 3-BP reacts with the cysteine moiety in glutathione (GSH) (4), the main ROS scavenger, leading to its depletion and increase of ROS levels (5) [19,21]. In addition, 3-BP can cause inhibition of the pentose phosphate pathway (PPP) due to hindrance of glucose-6-phosphate formation (6), which in turn can lead to a fall in NADPH and further GSH depletion. dNTP levels could be decreased and PPP is a major source of dNTP precursors (7) [77]. ROS can enter the nucleus and cause oxidation of DNA bases and DNA breaks (8) [78]. DNA damage can be exacerbated by smaller dNTP pools, which are crucial for DNA repair and cell survival [63,64].

3-BP exhibits broad activities, from anticancer [3,69], antifungal [65,70] to antibacterial [71] and anti-algal [72]. 3-BP, in the cell, acts pleiotropically by disrupting various processes (Figure 8). Previous studies were focused on the influence of 3-BP on cell metabolism, identification of the pathways of 3-BP uptake and extrusion and potential resistance mechanisms. The purpose of this work was to better understand the additional mechanism of 3-BP action by determining whether 3-BP is a genotoxic compound and thus whether 3-BP induces DNA damage. It seems that the 3-BP genotoxic activity demonstrated in this project is another mechanism with which to fight cancer cells, especially those in which the DNA repair process is disturbed.

## Figures and Tables

**Table 1:** Yeast strains used in this study.

Strain Name	Genotype	References
W303-1A	*MAT* **a** *ade2-1 can1-100 ura3-1 his3-11,15 leu2-3,112trp1-1 RAD5*	[48]
MC002	W303-1A, *rad51*Δ::*kanMX6*	[49]
MC006	W303-1A, *rad52*Δ::*kanMX6*	[49]
MC023	W303-1A, *rad59*Δ::*kan*MX6	[49]
N1-2B	W303-1A, *tel1*Δ::*URA3*	[50]
N1-3A	W303-1A, *mec1-1*	[50]
IL003	W303-1A, *apn1*Δ::*TRP1 apn2*Δ::*kanMX6*	[51]
IL006	W303-1A, *yku70*Δ::*kanMX6*	[51]
IL007	W303-1A, *yku70*Δ::*kanMX6 rad51*Δ::*TRP1*	[51]
W3749-14C	W303-1A, *ADE2 bar1*::*LEU2 RAD52-YFP*	[35]
W3775-12C	W303-1A, *ADE2 bar1*::*LEU2 RFA1-YFP*	[35]
W3483-10A	W303-1A, *ADE2 bar1*::*LEU2 MRE11-YFP*	[35]

## References

[1] Ko Y.H., Verhoeven H.A., Lee M.J., Corbin D.J., Vogl T.J., Pedersen P.L. (2012). A translational study “case report” on the small molecule “energy blocker” 3-bromopyruvate (3BP) as a potent anticancer agent: From bench side to bedside. J. Bioenerg. Biomembr..

[2] Majkowska-Skrobek G., Augustyniak D., Lis P., Bartkowiak A., Gonchar M., Ko Y.H., Pedersen P.L., Goffeau A., Ułaszewski S. (2014). Killing multiple myeloma cells with the small molecule 3-bromopyruvate: Implications for therapy. Anticancer Drugs.

[3] Ko Y.H., Niedźwiecka K., Casal M., Pedersen P.L., Ułaszewski S. (2018). 3-bromopyruvate (3BP) as a potent anticancer therapy in honor and memory of the late Professor Andre Goffeau. Yeast.

[4] Queirós O., Preto A., Pacheco A., Pinheiro C., Azevedo-Silva J., Moreira R., Pedro M., Ko Y.H., Pedersen P.L., Baltazar F. (2012). Butyrate activates the monocarboxylate transporter MCT4 expression in breast cancer cells and enhances the antitumor activity of 3-bromopyruvate. J. Bioenerg. Biomembr..

[5] Birsoy K., Wang T., Possemato R., Yilmaz O.H., Koch C.E., Chen W.W., Hutchins A.W., Gultekin Y., Peterson T.R., Carette J.E. (2013). MCT1 mediated transport of a toxic molecule is an effective strategy for targeting glycolytic tumors. Nat. Genet..

[6] Lis P., Zarzycki M., Ko Y.H., Casal M., Pedersen P.L., Goffeau A., Ułaszewski S. (2012). Transport and cytotoxicity of the anticancer drug 3-bromopyruvate in the yeast Saccharomyces cerevisiae. J. Bioenerg. Biomembr..

[7] Pedersen P.L., Mathupala S., Rempel A., Geschwind J.F., Ko Y.H. (2002). Mitochondrial bound type II hexokinase: A key player in the growth and survival of many cancers and an ideal prospect for therapeutic intervention. Biochim. Biophys. Acta..

[8] Mathupala S.P., Ko Y.H., Pedersen P.L. (2006). Hexokinase II: Cancer’s double-edged sword acting as both facilitator and gatekeeper of malignancy when bound to mitochondria. Oncogene.

[9] Mathupala S.P., Ko Y.H., Pedersen P.L. (2009). Hexokinase-2 bound to mitochondria: Cancer’s stygian link to the “Warburg effect” and a pivotal target for effective therapy. Semin. Cancer Biol..

[10] Ko Y.H., Smith B.L., Wang Y., Pomper M.G., Rini D.A., Torbenson M.S., Hullihen J., Pedersen P.L. (2004). Advanced cancers: Eradication in all cases using 3-bromopyruvate therapy to deplete ATP. Biochem. Biophys. Res. Commun..

[11] Ganapathy-Kanniappan S., Kunjithapatham R., Geschwind J.F. (2013). Anticancer efficacy of the metabolic blocker 3-bromopyruvate: Specific molecular targeting. Anticancer Res..

[12] Mulet C., Lederer F. (1977). Bromopyruvate as an affinity label for Baker’s Yeast flavocytochrome b_2_. Eur. J. Biochem..

[13] Macchioni L., Davidescu M., Roberti R., Corazzi L. (2014). The energy blockers 3-bromopyruvate and lonidamine: Effects on bioenergetics of brain mitochondria. J Bioenerg Biomembr..

[14] El Sayed S.M., El-Magd R.A., Shishido Y., Chung S.P., Sakai T., Watanabe H., Kagami S., Fukui K. (2012). D-amino acid oxidase gene therapy sensitizes glioma cells to the antiglycolytic effect of 3-bromopyruvate. Cancer Gene Ther..

[15] Ihrlund L.S., Hernlund E., Khan O., Shoshan M.C. (2008). 3-Bromopyruvate as inhibitor of tumour cell energy metabolism and chemopotentiator of platinum drugs. Mol. Oncol..

[16] Kim J.S., Ahn K.J., Kim J.A., Kim H.M., Lee J.D., Lee J.M., Kim S.J., Park J.H. (2008). Role of reactive oxygen species-mediated mitochondrial dysregulation in 3-bromopyruvate induced cell death in hepatoma cells. J. Bioenerg. Biomembr..

[17] Sun Y., Liu Z., Zou X., Lan Y., Sun X., Wang X., Zhao S., Jiand C., Liu H. (2015). Mechanisms underlying 3-bromopyruvate-induced cell death in colon cancer. J. Bioenerg. Biomembr..

[18] Sadowska-Bartosz I., Bartosz G. (2013). Effect of 3-bromopyruvic acid on human erythrocyte antioxidant defense system. Cell Biol. Int..

[19] Sadowska-Bartosz I., Szewczyk R., Jaremko L., Jaremko M., Bartosz G. (2016). Anticancer agent 3-bromopyruvic acid forms a conjugate with glutathione. Pharmacol. Rep..

[20] Niedźwiecka K., Dyląg M., Augustyniak D., Majkowska-Skrobek G., Cal-Bąkowska M., Ko Y.H., Ułaszewski S. (2016). Glutathione may have implications in the design of 3-bromopyruvate treatment protocols for both fungal and algal infections as well as multiple myeloma. Oncotarget.

[21] El Sayed S.M., Baghdadi H., Zolaly M., Almaramhy H.H., Ayat M., Donki J.G. (2017). The promising anticancer drug 3-bromopyruvate is metabolized through glutathione conjugation which affects chemoresistance and clinical practice: An evidence-based view. Med. Hypotheses.

[22] Hirschhaeuser F., Sattler U.G., Mueller-Klieser W. (2011). Lactate: A metabolic key player in cancer. Cancer Res..

[23] Pedersen P.L. (2012). 3-Bromopyruvate (3BP) a fast acting, promising, powerful, specific, and effective “small molecule” anti-cancer agent taken from labside to bedside: Introduction to a special issue. J. Bioenerg. Biomembr..

[24] Azevedo-Silva J., Queirós O., Baltazar F., Ułaszewski S., Goffeau A., Ko Y.H., Pedersen P.L., Preto A., Casal M. (2016). The anticancer agent 3-bromopyruvate: A simple but powerful molecule taken from the lab to the bedside. J. Bioenerg. Biomembr..

[25] Ko Y.H., Pedersen P.L., Geschwind J.F. (2001). Glucose catabolism in the rabbit VX2 tumor model for liver cancer: Characterization and targeting hexokinase. Cancer Lett..

[26] Dyląg M., Lis P., Niedźwiecka K., Ko Y.H., Pedersen P.L., Goffeau A., Ułaszewski S. (2013). 3-bromopyruvate: A novel antifungal agent against the human pathogen Cryptococcus neoformans. Biochem. Biophys. Res. Commun..

[27] Sadowska-Bartosz I., Soszyński M., Ułaszewski S., Ko Y.H., Bartosz G. (2014). Transport of 3-bromopyruvate across the human erythrocyte membrane. Cell Mol. Biol. Lett..

[28] Wang W., Daley J.M., Kwon Y., Krasner D.S., Sung P. (2017). Plasticity of the Mre11-Rad50-Xrs2-Sae2 nuclease ensemble in the processing of DNA bound obstacles. Genes Dev..

[29] Moore J.D., Yazgan O., Ataian Y., Krebs J.E. (2007). Diverse roles for histone H2A modifications in DNA damage response pathways in yeast. Genetics.

[30] Lanz M.C., Dibitetto D., Smolka M.B. (2019). DNA damage kinase signaling: Checkpoint and repair at 30 years. Embo J..

[31] Xue C., Wang W., Crickard J.B., Moevus C.J., Kwon Y., Sung P., Greene E.C. (2019). Regulatory control of Sgs1 and Dna2 during eukaryotic DNA end resection. Proc. Natl. Acad. Sci. USA.

[32] David S.S., O’Shea V.L., Kundu S. (2007). Base-excision repair of oxidative DNA damage. Nature.

[33] De Laat W.L., Jaspers N.G., Hoeijmakers J.H. (1999). Molecular mechanism of nucleotide excision repair. Genes Dev..

[34] Lieber M.R. (2010). The mechanism of double-strand DNA break repair by the nonhomologous DNA end-joining pathway. Annu. Rev. Biochem..

[35] Lisby M., Barlow J.H., Burgess R.C., Rothstein R. (2004). Choreography of the DNA damage response: Spatiotemporal relationships among checkpoint and repair proteins. Cell.

[36] Ira G., Pellicioli A., Balijja A., Wang X., Fiorani S., Carotenuto W., Liberi G., Bressan D., Wan L., Hollingsworth N.M. (2004). DNA end resection, homologous recombination and DNA damage checkpoint activation require CDK1. Nature.

[37] Bai Y., Symington L.S. (1996). A Rad52 homolog is required for RAD51-independent mitotic recombination in Saccharomyces cerevisiae. Genes Dev..

[38] Chen Q., Ijpma A., Greider C.W. (2001). Two survivor pathways that allow growth in the absence of telomerase are generated by distinct telomere recombination events. Mol. Cell Biol..

[39] Song B., Sung P. (2000). Functional interactions among yeast Rad51 recombinase, Rad52 mediator, and replication protein A in DNA strand exchange. J. Biol. Chem..

[40] Feng Q., Düring L., de Mayolo A.A., Lettier G., Lisby M., Erdeniz N., Mortensen U.H., Rothstein R. (2007). Rad52 and Rad59 exhibit both overlapping and distinct functions. DNA Repair (Amst).

[41] Hasegawa T., Takahashi J., Nagasawa S., Doi M., Moriyama A., Iwahashi H. (2020). DNA Strand Break Properties of Protoporphyrin IX by X-ray Irradiation against Melanoma. Int. J. Mol. Sci..

[42] Lee T.H., Kang T.H. (2019). DNA Oxidation and Excision Repair Pathways. Int. J. Mol. Sci..

[43] Duthie S.J., Collins A.R., Duthie G.G., Dobson V.L. (1997). Quercetin and myricetin protect against hydrogen ž peroxide-induced DNA damage strand breaks and oxidised/pyrimidines in human lymphocytes. Mutat Res..

[44] Srinivas U.S., Tan B.W.Q., Vellayappan B.A., Jeyasekharan A.D. (2019). ROS and the DNA damage response in cancer. Redox Biol..

[45] Sharma V., Collins L.B., Chen T.H., Herr N., Takeda S., Sun W., Swenberg J.A., Nakamura J. (2016). Oxidative stress at low levels can induce clustered DNA lesions leading to NHEJ mediated mutations. Oncotarget..

[46] Akhtar S., Najafzadeh M., Isreb M., Newton L., Gopalan R.C., Anderson D. (2020). ROS-induced oxidative damage in lymphocytes ex vivo/in vitro from healthy individuals and MGUS patients: Protection by myricetin bulk and nanoforms. Arch Toxicol..

[47] Boiteux S., Gellon L., Guibourt N. (2002). Repair of 8-oxoguanine in Saccharomyces cerevisiae: Interplay of DNA repair and replication mechanisms. Free Radic. Biol. Med..

[48] Thomas B.J., Rothstein R. (1989). Elevated recombination rates in transcriptionally active DNA. Cell.

[49] Cal-Bakowska M., Litwin I., Bocer T., Wysocki R., Dziadkowiec D. (2011). The Swi2-Snf2-like protein Uls1 is involved in replication stress response. Nucleic Acids Res..

[50] Redon C., Pilch D.R., Rogakou E.P., Orr A.N., Lowndes N.F., Bonner W.M. (2003). Yeast Histone 2A serine 129 is essential for the efficient repair of checkpoint-blind DNA damage. EMBO Rep..

[51] Litwin I., Bocer T., Dziadkowiec D., Wysocki R. (2013). Oxidative stress and replication-independent DNA breakage induced by arsenic in *Saccharomyces cerevisiae*. PLoS Genet..

[52] Litwin I., Bakowski T., Szakal B., Pilarczyk E., Maciaszczyk-Dziubińska E., Branzei D., Wysocki R. (2018). Error-free DNA damage tolerance pathway is facilitated by the Irc5 translocase through cohesin. Embo J..

[53] Downs J.A., Lowndes N.F., Jackson S.P. (2000). A role for Saccharomyces cerevisiae histone H2A in DNA repair. Nature.

[54] Shroff R., Arbel-Eden A., Pilch D., Ira G., Bonner W.M., Petrini J.H., Haber J.E., Lichten M. (2004). Distribution and dynamics of chromatin modification induced by a defined DNA double-strand break. Curr. Biol..

[55] Leroy C., Mann C., Marsolier M.C. (2001). Silent repair accounts for cell cycle specificity in the signaling of oxidative DNA lesions. Embo J..

[56] Lisby M., Rothstein R. (2009). Choreography of recombination proteins during the DNA damage response. DNA repair (Amst).

[57] Rogakou E.P., Pilch D.R., Orr A.H., Ivanova V.S., Bonner W.M. (1998). DNA double-stranded breaks induce histone H2AX phosphorylation on serine 139. J. Biol. Chem..

[58] Ward I.M., Chen J. (2001). Histone H2AX is phosphorylated in an ATR-dependent manner in response to replicational stress. J. Biol. Chem..

[59] Rogakou E.P., Boon C., Redon C., Bonner W.M. (1999). Megabase chromatin domains involved in DNA double-strand breaks in vivo. J. Cell Biol..

[60] Rai Y., Yadav P., Kumari N., Kalra N., Bhatt A.N. (2019). Hexokinase II inhibition by 3-bromopyruvate sensitizes myeloid leukemic cells K-562 to anti-leukemic drug, daunorubicin. Biosci. Rep..

[61] Zhao H., Tanaka T., Halicka H.D., Traganos F., Zarebski M., Dobrucki J., Darzynkiewicz Z. (2007). Cytometric assessment of DNA damage by exogenous and endogenous oxidants reports aging-related processes. Cytometry A..

[62] Spector D., Labarre J., Toledano M.B. (2001). A genetic investigation of the essential role of glutathione: Mutations in the proline biosynthesis pathway are the only suppressors of glutathione auxotrophy in yeast. J. Biol. Chem..

[63] Chabes A., Georgieva B., Domkin V., Zhao X., Rothstein R., Thelander L. (2003). Survival of DNA damage in yeast directly depends on increased dNTP levels allowed by relaxed feedback inhibition of ribonucleotide reductase. Cell.

[64] Kohnken R., Kodigepalli K.M., Wu L. (2015). Regulation of deoxynucleotide metabolism in cancer: Novel mechanisms and therapeutic implications. Mol. Cancer.

[65] Ferreira da Silva F., do N Varella M.T., Jones N.C., Vrønning Hoffmann S., Denifl S., Bald I., Kopyra J. (2019). Electron-induced reactions in 3-bromopyruvic acid. Chemistry.

[66] Dizdaroglu M., Jaruga P., Birincioglu M., Rodriguez H. (2002). Free radical-induced damage to DNA: Mechanisms and measurement. Free Radic. Biol. Med..

[67] Letavayová L., Marková E., Hermanská K., Vlcková V., Vlasáková D., Chovanec M., Brozmanová J. (2006). Relative contribution of homologous recombination and non-homologous end-joining to DNA double-strand break repair after oxidative stress in Saccharomyces cerevisiae. DNA Repair (Amst)..

[68] Chen J., Ghorai M.K., Kenney G., Stubbe J. (2008). Mechanistic studies on bleomycin-mediated DNA damage: Multiple binding modes can result in double-stranded DNA cleavage. Nucleic. Acids Res..

[69] Lis P., Dyląg M., Przywara K., Ko Y., Pedersen P.L., Goffeau A., Ułaszewski S. (2016). The HK2 Dependent “Warburg Effect” and mitochondrial oxidative phosphorylation in cancer: Targets for effective therapy with 3-Bromopyruvate. Molecules.

[70] Dyląg M., Lis P., Ko Y.H., Pedersen P.L., Goffeau A., Ułaszewski S. (2012). Use of the composition of 3-bromopyruvate as a second application of a medicament for the treatment of fungal infections. Patent.

[71] Visca P., Pisa F., Imperi F. (2018). The antimetabolite 3-bromopyruvate selectively inhibits *Staphylococcus aureus*. Int. J. Antimicrob. Agents..

[72] Jagielski T., Niedźwiecka K., Roeske K., Dyląg M. (2018). 3-Bromopyruvate as an alternative option for the treatment of Protothecosis. Front Pharmacol..

[73] Fan T., Sun G., Sun X., Zhao L., Zhong R., Peng Y. (2019). Tumor energy metabolism and potential of 3-Bromopyruvate as an inhibitor of aerobic glycolysis: Implications in tumor treatment. Cancers.

[74] Jardim-Messeder D., Moreira-Pacheco F. (2016). 3-Bromopyruvic acid inhibits tricarboxylic acid cycle and glutaminolysis in HepG2 cells. Anticancer Res..

[75] Slane B.G., Aykin-Burns N., Smith B.J., Kalen A.L., Goswami P.C., Domann F.E., Spitz D.R. (2006). Mutation of succinate dehydrogenase subunit C results in increased O^2−^, oxidative stress, and genomic instability. Cancer Res..

[76] Tretter L., Patocs A., Chinopoulos C. (2016). Succinate, an intermediate in metabolism, signal transduction, ROS, hypoxia, and tumorigenesis. Biochim. Biophys. Acta.

[77] Jin L., Zhou Y. (2019). Crucial role of the pentose phosphate pathway in malignant tumors. Oncol. Lett..

[78] Yu T.W., Anderson D. (1997). Reactive oxygen species-induced DNA damage and its modification: A chemical investigation. Mutat. Res..

